# Helping or controlling? A multi-method case study of a mother-daughter dyad and their interactions during trauma-focused therapy

**DOI:** 10.1080/20008066.2026.2731853

**Published:** 2026-09-28

**Authors:** Kyra E. Verboon, Marie-Louise J. Kullberg, Bart Verkuil, Peter Muris, A. Bexkens, Bernet M. Elzinga

**Affiliations:** a Department of Clinical Psychology, Leiden University, Leiden, The Netherlands; b Department of Clinical Psychological Science, Faculty of Psychology and Neuroscience, Maastricht University, Maastricht, The Netherlands; c Youz-Parnassia Group, Maastricht, The Netherlands; d Department of Developmental and Educational Psychology, Leiden University, Leiden, The Netherlands; e GGZ Delfland, Delft, The Netherlands

**Keywords:** Parent-adolescent interaction, trauma-focused therapy, multi-method assessment, multi-informant perspectives, ecological momentary assessments (EMA), overprotection, Interacción padre-adolescente, terapia centrada en el trauma, sobreprotección, enfoque de múltiples métodos, perspectivas de múltiples informantes, evaluaciones ecológicas momentáneas (EEM)

## Abstract

**Background:** Parents play a pivotal role in supporting children following trauma, yet little is known about daily parent-child interactions during trauma-focused treatment.

**Objective:** This study aimed to examine daily dynamics related to parental overprotective and anxious rearing behaviours and adolescent PTSD symptoms during intensive trauma-focused therapy, and to evaluate the added value of a multi-method, multi-informant assessment approach.

**Method:** A mother-daughter dyad, including a 14-year-old adolescent undergoing intensive trauma-focused therapy, was assessed over a five-week period. Data collection included baseline and follow-up questionnaires, ecological momentary assessment (EMA; four prompts per day), observational assessments, and qualitative interviews enabling the capture of multiple perspectives on parenting behaviours and adolescent PTSD symptoms.

**Results:** Within this mother-daughter dyad, EMA captured substantial day-to-day fluctuations in both maternal behaviour and PTSD symptoms, and revealed reciprocal associations between maternal overprotection and adolescent PTSD symptoms. Both were not visible in retrospective surveys. Integrating EMA findings with observational and qualitative data yielded additional insight into maternal intentions, contextual influences, and discrepancies between mother and daughter perspectives.

**Conclusions:** This case study illustrates the added value of multi-method, multi-informant approaches to capture clinically relevant parent-child dynamics during trauma-focused therapy. Although these approaches may require greater effort from families and clinicians than single-method assessments, they offer richer and more nuanced insights into parent-child dynamics in daily life. Future research should focus on developing more efficient assessment strategies, for example by using brief EMA protocols or integrating EMA in clinical care.

## HIGHLIGHTS

- Assessing both parents and adolescents is crucial for a systemic understanding of adolescent psychotraumatology and for informing personalised clinical interventions.
- Combining ecological momentary assessments with observations and interviews provides a detailed view of daily parent-child interactions and their relation to adolescent PTSD symptoms.
- Ecological momentary assessments during trauma-focused therapy is valuable for capturing symptom fluctuations and examining reciprocal associations between parental overprotection and adolescent symptoms.

## Abstract (es)

**Antecedente:** Los padres desempeñan un papel fundamental al dar soporte a los niños luego de un trauma; no obstante, poco se conoce sobre las interacciones diarias entre padres e hijos durante la terapia centrada en el trauma.

**Objetivo:** Este estudio busca evaluar las dinámicas diarias asociadas a conductas de crianza sobreprotectoras o ansiosas y los síntomas del trastorno de estrés postraumático (TEPT) en adolescentes durante la terapia intensiva centrada en el trauma y, además, evaluar el valor agregado de los enfoques de múltiples métodos y con múltiples informantes.

**Métodos:** Se evalúo una diada madre-hija, que incluía a una adolescente de 14 años recibiendo terapia intensiva centrada en el trauma, durante un periodo de cinco semanas. La recolección de datos incluyó cuestionarios iniciales y de seguimiento, evaluaciones ecológicas momentáneas (EEM; cuatro indicaciones diarias), evaluaciones observacionales y entrevistas cualitativas que permitían captar múltiples perspectivas en las conductas parentales y en los síntomas del TEPT en la adolescente.

**Resultados:** Dentro de esta diada madre-hija, las EEM captaron fluctuaciones sustanciales diarias tanto en las conductas maternas como en los síntomas del TEPT, además de revelar asociaciones recíprocas entre la sobreprotección materna y los síntomas del TEPT en la adolescente. Ninguno de estos dos hallazgos pudo ser detectado en las evaluaciones retrospectivas. El integrar los hallazgos de las EEM con datos observacionales y cualitativos brindaron una mejor comprensión de las intenciones maternas, influencias del contexto y discrepancias entre las perspectivas de la madre y de la hija.

**Conclusiones:** Este estudio de caso ilustra el valor agregado de enfoques de múltiples métodos y con múltiples informantes para captar dinámicas clínicamente relevantes entre padres e hijos durante la terapia centrada en el trauma. Pese a que estos enfoques puedan requerir mayor esfuerzo por parte de la familia y los clínicos que las evaluaciones de un solo método, ofrecen una comprensión más profunda y matizada de las dinámicas de la vida diaria entre padres e hijos. Las futuras investigaciones deberían enfocarse en desarrollar estrategias de evaluación más eficientes como, por ejemplo, el emplear protocolos breves de EEM o integrar las EEM en la atención clínica.

## 1. Introduction

Although parents play a crucial role in supporting children after trauma and are increasingly involved in treatment, empirical research still offers limited insight into parent-child interactions within real-time clinical contexts (Afzal et al., 2023; Williamson et al., 2017). Most studies rely on cross-sectional, single-informant surveys conducted outside therapeutic settings. Yet, family dynamics and perceptions of parenting can vary considerably – *between* and *within* families, and *across* contexts such as everyday life and therapy (e.g. De Los Reyes et al., 2019; Janssen, Elzinga, et al., 2021). This variability constrains the ability of current research to identify which parenting behaviours facilitate or hinder adolescents’ coping with trauma-related symptoms in their unique family environments. To better capture these dynamic processes, research must include precise assessments of parent-child interactions in daily life, incorporate both parents’ and children’s perspectives, and employ measures that validate subjective reports. In this study, we examine a single mother-daughter dyad before, during, and after the child’s trauma-focused therapy to illustrate how multiple methods and perspectives can yield distinct yet complementary insights into daily interactions. Focusing on the interplay between adolescent posttraumatic stress disorder (PTSD) symptoms and parental overprotection and anxious rearing, we explore how different measures converge and discuss their implications for both research and clinical practice.

### 1.1. The crucial role of parents in the aftermath of trauma

Many children experience traumatic events such as accidents, abuse, or the sudden loss of a loved one (McLaughlin et al., 2013). While most display transient anxiety and stress reactions, some develop enduring psychological difficulties, including PTSD and other anxiety-related disorders (Alisic et al., 2014; McLaughlin et al., 2013). A growing body of research highlights the pivotal role of parents in shaping children’s psychological adjustment following trauma exposure (e.g. Cobham et al., 2016; Williamson et al., 2017). Supportive and caring parenting can buffer against stress-related symptoms (Lima et al., 2014), whereas hostile, critical, or rejecting parenting has been linked to heightened symptom severity in children (Williamson et al., 2017). Even well-intentioned behaviours aimed at protecting the child – such as parental overprotection (excessive involvement in the child’s activities) and anxious rearing (frequent expressions of worry) – may inadvertently maintain or exacerbate anxiety, depression, and PTSD symptoms in youth (Afzal et al., 2023; Grüner et al., 1999; Vigdal & Brønnick, 2022; Williamson et al., 2017). These behaviours are particularly interesting to study because they represent a paradoxical form of parenting: although motivated by care and concern, they may unintentionally contribute to the maintenance of psychological symptoms.

Building on evidence that parental support alleviates children’s trauma-related symptoms, caregivers are increasingly integrated into trauma-focused therapies, often through parent trainings conducted alongside the child’s therapy (Gigengack et al., 2019). These trainings provide psychoeducation about PTSD and coping, and include self-reflection exercises to improve parents’ understanding of, and responsiveness to, their child’s needs. Such interventions increase parental trauma sensitivity and reduce both parental stress and children’s symptoms (Gigengack et al., 2019; Konijn et al., 2020). Although these outcomes are promising, little is known about how changes in parents’ everyday behaviours – potentially resulting from such training – translate into improvements in children’s symptoms. This gap underscores the need for practice-oriented, ecologically valid assessments that better connect research findings with clinical practice.

### 1.2. Capturing daily family dynamics with ecological momentary assessment

To examine the dynamic, family-specific nature of parent-child interactions, intensive repeated-measures designs such as ecological momentary assessment (EMA) are particularly valuable. Traditional cross-sectional surveys capture only average patterns and cannot determine the directionality of effects (e.g. whether parenting influences symptoms or vice versa) nor do they reflect how parent-child interactions unfold in real-life contexts. In contrast, EMA captures real-time interactions, minimises recall bias, and reveals the nuanced day-to-day dynamics of parent-child relationships, including those occurring during therapy (Keijsers et al., 2022). Recent research has increasingly employed EMA to track daily fluctuations in PTSD symptoms among adults (Chun, 2016) and to investigate parenting in relation to adolescent affect and depressive symptoms (Bülow et al., 2022; Janssen, Elzinga, et al., 2021; Veenman et al., 2024). However, despite its promise, EMA research on daily parent–child interactions following trauma remains scarce, particularly with regard to overprotective parenting and studies conducted within clinical settings.

### 1.3. Parent-child discrepancies in reported parenting behaviours

When examining parenting behaviours, it is crucial to consider who reports them. Previous reviews indicate that most studies rely on either parent or child reports (Afzal et al., 2023; Williamson et al., 2017), even though parents and adolescents often perceive parenting differently (De Los Reyes et al., 2019; Hou et al., 2020). Parents typically rate their own behaviour more positively, describing themselves as warmer and less critical than their children do. Similarly, mothers often view themselves as less controlling than their adolescents perceive them to be (Bögels & van Melick, 2004). Such parent-child discrepancies – for example, when adolescents perceive more parental overprotection than their mothers report – have been linked to poorer adolescent mental health (Janssen, Verkuil, et al., 2021; Van Petegem et al., 2020; Wang & Hawk, 2023). This highlights the importance of considering both perspectives and their (dis)agreements. Although a few studies have examined daily parent-child discrepancies (Janssen, Verkuil, et al., 2021; Xu & Zheng, 2022), daily discrepancies in parental overprotection during trauma-focused therapy, and their association with PTSD symptoms, remain unexplored. Incorporating both parent and adolescent perspectives into EMA designs can yield a more nuanced, ecologically valid understanding of daily parenting dynamics throughout therapy.

### 1.4. Complementary assessments: observations and interviews

Although incorporating EMA and multiple informants enhances understanding of daily parenting dynamics, certain limitations remain. EMA can capture contextual details such as location or social company (Keijsers et al., 2022), yet its close-ended format often omits broader contextual nuances. Moreover, as a self-report method, EMA is subject to social desirability biases and depends on participants’ self-awareness, thus capturing only what participants consciously notice and choose to report (Schwarz, 2007). Complementary assessments, including observations and qualitative interviews, can address these limitations by offering additional perspectives. Such methods enable researchers and clinicians to interpret daily EMA data within the broader therapeutic and family context and to validate or refine self-reported information. Integrating multiple data sources provides a more comprehensive and ecologically grounded understanding of parent-child interactions.

### 1.5. The present study

This exploratory study employs a multi-method design to investigate daily parent-child interactions within a single mother-daughter dyad undergoing trauma-focused therapy. Maternal overprotection and anxious rearing are assessed from both the mother’s and the daughter’s perspectives using surveys, EMA, and interviews, while researcher observations offer an external viewpoint of their interactions. Collectively, these complementary methods provide a rich and nuanced understanding of parenting dynamics and illustrate the value of integrating multiple approaches to inform both research and clinical practice.

## 2. Methods

### 2.1. Participants

This study examined EMA data from a 14-year-old girl and her 52-year-old biological mother, who participated in a one-week intensive trauma-focused treatment programme (i-TFT) at a Dutch mental health institution. This dyad was selected for detailed analysis because of their exceptionally high EMA compliance (>90%), which was substantially higher than that of most other participants (15–50%). Adolescents were eligible for inclusion if they participated in the i-TFT, were 10–18 years old, lived with the participating parent at least 90% of the time, and had sufficient Dutch proficiency. Parents were required to have a good command of Dutch.

All participants who received i-TFT were screened for eligibility to participate in the study. A total of 20 adolescents underwent i-TFT during four different weeks over a two-year period. Five adolescents agreed to participate in the study, alongside four mothers. When fathers were present, they were also invited to participate; however, none agreed for reasons that remain unknown. Of the remaining 15 adolescents, four did not meet the inclusion criteria because they were either not living at home or lacked proficiency in Dutch. Among the remaining eligible adolescents, some enrolled in the i-TFT at the last minute, leaving insufficient time to complete the baseline EMA before treatment began. In other cases, practitioners considered participation too burdensome given the vulnerability of the families, or families did not respond to telephone calls or emails.

### 2.2. Intensive trauma-focused treatment

The i-TFT is a brief, intensive trauma-focused treatment programme provided as an adjunct to standard PTSD treatment, with the aim of further reducing PTSD symptoms in adolescents. The programme was delivered within one week at a Dutch mental health institution and consisted of four treatment days (Monday, Tuesday, Thursday, and Friday). All adolescents between the ages of 10 and 18 years who were experiencing PTSD symptoms and received treatment for these symptoms were eligible to participate, with a maximum of six adolescents per week. Adolescents received two hours of individual therapy per day: one hour of exposure therapy and one hour of Eye Movement Desensitization and Reprocessing (EMDR). Each day, adolescents selected a trauma-related trigger that was addressed by a different licensed psychologist. If adolescents participated in the programme, their parents were invited to participate in a concurrent group parent training during the same week. A detailed overview of the daily schedule is provided in Supplementary Materials S1. Parents participated in the Dutch adaption of the psychoeducation programme ‘Caring for Children Who Have Experienced Trauma’ (Coppens & van Kregten, 2015). The training, delivered by two licensed psychologists, covered topics such as establishing safety, maintaining calmness as a co-regulator during distress, and managing parental hypervigilance to foster a supportive environment. Parents also engaged in practical exercises encouraging reflection on their own and their child’s behaviour, promoting awareness of the child’s experiences and their impact on family. The modules of each session are described in the Supplementary Materials S1. In addition to the therapeutic sessions, families participated in activities designed to promote social interaction among participants. These included brief end-of-day reflection sessions between adolescents and their parents, as well as 30 min of physical activity involving all participating families. During the study period, five families took part in the i-TFT.

### 2.3. Questionnaires

#### 2.3.1. PTSD symptoms

PTSD symptoms were assessed at baseline and follow-up using the Dutch version of the Child and Adolescent Trauma Screener 2 (CATS-2; Sachser et al., 2022), the *Kind en Jeugd Trauma Screener* (KJTS; Kooij et al., 2025). The KJTS is a self-report questionnaire that evaluates exposure to potentially traumatic events and current PTSD symptoms. It consists of three parts: The first part includes 15 items assessing whether participants experienced potentially traumatic events (e.g. death of a significant person, sexual abuse), with binary ‘yes’/‘no’ responses. The second part comprises 20 items assessing PTSD symptoms related to the most distressing traumatic event, rated on a 4-point Likert scale (0 = *Never* to 3 = *Almost always*) reflecting symptom frequency over the past two weeks. The third part includes five items measuring deficits in psychosocial functioning. The KJTS has demonstrated good reliability (Intrusions: *α* = .86; Avoidance: *α* = .68, Negative mood and cognition: *α* = .86; Arousal: *α* = .72) and validity (Kooij et al., 2025).

#### 2.3.2. Parenting behaviours

Parenting behaviours were assessed using the Dutch version of the Egna Minnen Beträffande Uppfostran (EMBU), including four 10-item subscales – rejection, emotional warmth, overprotection, and anxious rearing (Muris et al., 2003). Items were rated on a 4-point Likert scale (1 = *Never* to 4 = *Always*), and subscale scores were computed by summing the item responses. For the purpose of this study, the overprotection and anxious rearing subscales were used to assess adolescents’ perceptions of their parents’ overprotective and anxious rearing behaviours. Example items are ‘My parent watches me carefully’ (overprotection) and ‘My parent is worried when he/she doesn’t know what I am doing’ (anxious rearing). The EMBU was administered at baseline and follow-up, with adolescents reporting on the parenting behaviours of the participating parent. The overprotection and anxious rearing subscales show adequate internal consistency and sufficient reliability (α’s ranging from .66 to .79; Muris et al., 2003).

### 2.4. EMA

#### 2.4.1. PTSD symptoms

Daily PTSD symptoms were assessed via EMA using eight items from the KJTS, based on the eight-item version of the PTSD Checklist for DSM-5 (PCL-5; Price et al., 2016). At each prompt, the adolescent indicated whether she had experienced any PTSD symptoms since the previous beep. Each symptom was assessed with two items rated on a 4-point Likert scale (0 = *Never* to 3 = *Almost always*), which were summed to generate a symptom score. The assessed symptoms covered four domains: intrusions (e.g. involuntary distressing thoughts), avoidance (e.g. avoiding trauma-related reminders), negative alterations in mood and cognition (e.g. negative beliefs about oneself or the world), and hyperarousal (e.g. being easily startled, difficulty concentrating).

#### 2.4.2. Parenting behaviours

Daily parenting behaviour was assessed by asking the adolescent and the mother whether they had interacted since the previous prompt. If so, both rated overprotective and anxious parenting using three items on a 7-point Likert scale (1 = *Not at all* to 7 = *Very much*): (1) ‘My mother tried to help me even though this was not necessary’ (unnecessary help), (2) ‘My mother kept a close eye on me’ (excessive monitoring), and (3) ‘My mother warned me about possible dangers’ (cautious warning). The mother completed parallel first-person items (i.e. ‘I tried to help … ’). The items were adapted from EMA measures developed by Luijk et al. (2024) and the EMBU overprotection subscale (Muris et al., 2003).

### 2.5. Procedures

The study was conducted at a specialised Dutch mental health institution during the i-TFT for adolescents and their parents. The i-TFT was offered as a supplementary intervention to participants’ regular treatment. Upon registration, adolescents and their parents were informed about the study by their therapist. If families agreed to receive more information, the researcher contacted them by telephone and email to provide further details. Families were then given one week to decide whether to participate in the study. Adolescents could participate either individually or together with one or both parents. Families who agreed to participate met with the researcher to provide written informed consent. For adolescents aged 12–15 years, consent was obtained from both parents or legal guardians, whereas for those aged 16 years or older, the adolescent’s own written consent was sufficient conform Dutch regulations. Figure 1 illustrates the timeline of the study phases and assessment schedule.

**Figure 1.**
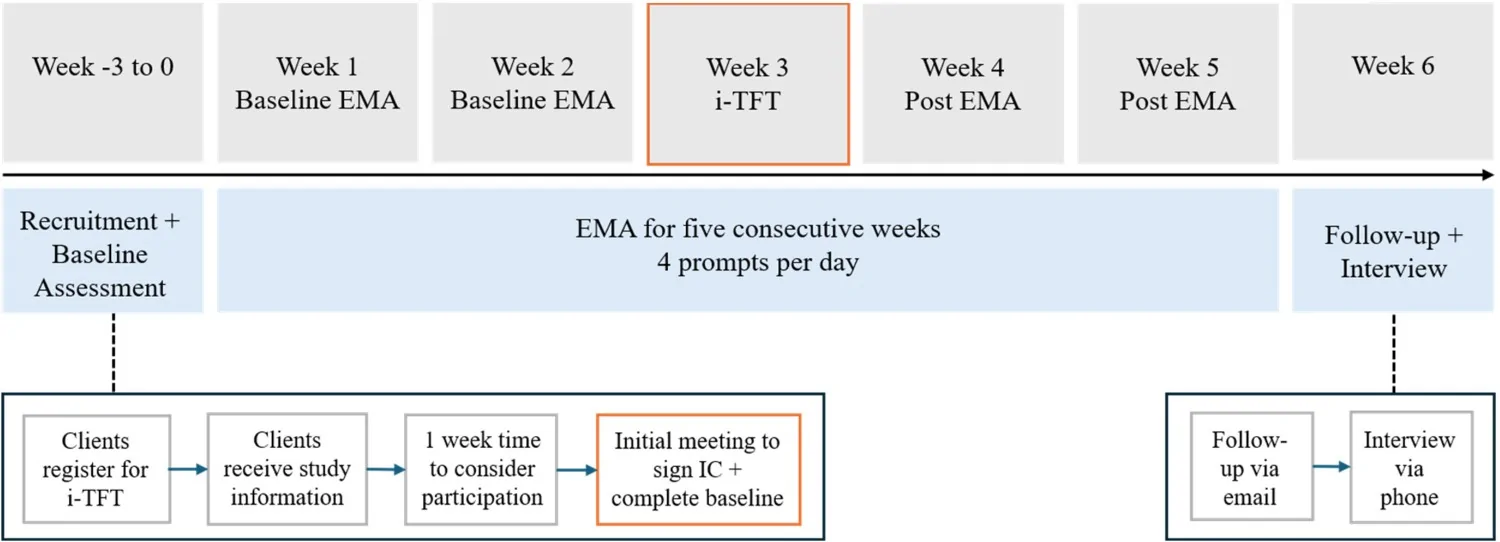
Study timeline showing recruitment, baseline, EMA and follow-up assessments. Note: The orange-outlined boxes indicate the time points at which observations were made.

Baseline questionnaires were completed during an initial face-to-face session, which also included instructions for installing the *m-Path* application and scheduling assessment times. Follow-up questionnaires were completed after the EMA period. The EMA period lasted five weeks (35 days), beginning two weeks before the i-TFT, encompassing the i-TFT, and continuing for two weeks afterward. Both the adolescent and the mother received four EMA prompts per day, scheduled at approximately equal intervals between 7:00 am and 11:00 pm, accessible for 1–3 h, and requiring on average four minutes to complete. The adolescent completed 90% of the EMA prompts over 34 days, and the mother completed 91% over 35 days. Missing data were not imputed, as doing so could compromise data validity, particularly since some missingness reflected no contact between mother and adolescent rather than non-response. For descriptive analyses, daily averages were computed, while correlations were based solely on complete observations.

During the baseline assessment and the i-TFT, the researcher conducted naturalistic observations of mother-daughter interactions. These observations took place within the clinical setting during moments when the mother and adolescent were together, without being assigned a predefined task. The researcher recorded field notes on parent-adolescent interactions, focusing on behavioural dynamics and contextual factors. Observations were not systematically coded using a standardised coding scheme, as the aim was to gather contextual information rather than to quantify parenting behaviour.

Following the EMA period, semi-structured interviews were conducted separately with the adolescent and the mother. The interviews explored participants’ experiences with the EMA procedure and the treatment, as well as perceived changes in behaviour, emotions, and parent-child interactions over the preceding weeks.

Participants received compensation of up to €45, depending on the number and consistency of completed assessments. To enhance engagement, they also earned virtual incentives for daily participation, consecutive streaks, and achievement of predefined milestones.

Written informed consent for the use of the data in this publication was obtained from both the parent and the child. In consultation with the institutional privacy officer, it was determined that participants would not review the manuscript to avoid inadvertent disclosure of information between family members. All potentially identifiable information has been altered or omitted to ensure anonymity. The study was approved by the Psychology Research Ethics Committee of Leiden University, the Netherlands (2023-04-14-B.M. Elzinga-V2-4419).

### 2.6. Statistical analyses

All analyses were conducted in RStudio (version 2024.09.0; R version 4.4.1). First, to provide a general, descriptive overview of day-to-day fluctuations in symptoms and parenting behaviours, time-series plots displaying daily means were generated, together with average scores and standard deviations for each study period (pre-, during, and post-i-TFT). Regression analyses were performed to test mean differences across periods, linear time trends within each period, and differences in slopes between periods. Second, discrepancies between parent- and adolescent-reported parenting behaviours were evaluated using Spearman correlations between corresponding items, difference scores (parent minus adolescent reports), and their associations with PTSD symptoms. Mean difference scores were also compared across periods to assess whether discrepancies decreased following i-TFT. Third, bidirectional associations between PTSD symptoms and parenting were explored using autocorrelations, contemporaneous correlations, and cross-lagged correlations based on the four momentary assessments per day. Specifically, analyses examined whether parenting behaviours at time *t* predicted PTSD symptoms at time *t* *+* 1, and vice versa.

## 3. Results

### 3.1. Case description

The adolescent was a 14-year-old Dutch girl living with her biological parents and two siblings, who attended senior general secondary education (in Dutch: *HAVO*). She had experienced (threats of) physical abuse by her father, which had affected herself and other family members. Although she also reported incidents of sexual abuse outside the family, the experience with physical abuse within the family had the most profound psychological impact. The girl was receiving trauma-focused treatment for PTSD and participated in the i-TFT with both parents as a supplement. At baseline, she reported clinically significant PTSD symptoms on the KJTS (score = 41), indicating severe trauma-related distress, and met the DSM-5 criteria for PTSD. Two weeks after the i-TFT, her total PTSD symptom severity had decreased (score = 28), reflecting a reduction in trauma-related distress. She continued to meet symptom criteria for intrusions, arousal, and negative mood and cognitions, but no longer for avoidance; therefore, she no longer met full DSM-5 criteria for PTSD at follow-up (Supplementary Table S1).

Regarding perceived maternal parenting, the adolescent reported elevated baseline scores on maternal overprotection (27) and anxious rearing (36). Compared to a large normative adolescent sample (*N* = 1681, *M_age_* = 12.95, *SD_age_* = 1.67, range 9–17; Muris et al., 2003), in which mothers scored an average of 20.88 (*SD* = 3.82) on overprotection and 21.99 (*SD* = 4.73) on anxious rearing, her scores were substantially higher (Figure 2). The interaction between mother and daughter during the first appointment (illustrated in Box 1) exemplified the daughter’s perception of high maternal control, consistent with her response of ‘often’ to the EMBU item: ‘Your mother thinks she should decide everything for you’. At follow-up, both overprotection and anxious rearing scores decreased (to 24 and 28, respectively). Although both scores remained higher than the normative mean, the overprotection score fell within the normative 95% confidence interval, suggesting that it was not meaningfully elevated relative to normative levels.

Box 1. Early dynamics

The mother and daughter visited the mental health institution to complete baseline questionnaires, install the m-path application, and schedule assessments. When asked about the timing of the first daily assessment, the daughter suggested a preferred time. The mother reacted immediately by mentioning that the assessment should take place 30 min earlier so they could complete it together before breakfast.

**Figure 2.**
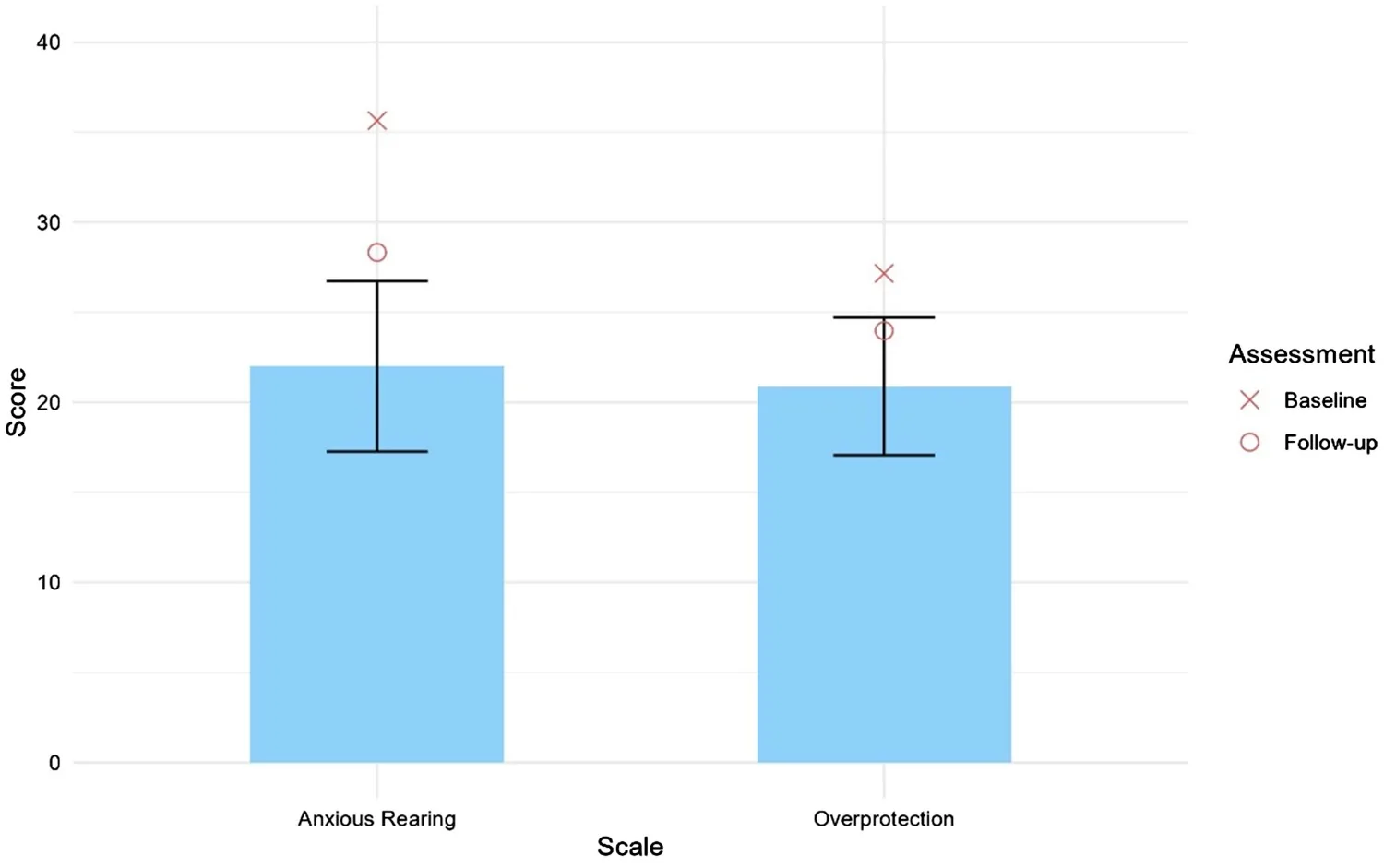
Maternal anxious rearing and overprotection scores of the girl in comparison to the norm group. Note: Blue bars show norm group means (and standard deviations) based on Muris et al. (2003). The cross and dot indicate the adolescent’s scores at baseline and follow-up, respectively, reflecting her perceptions of her mother’s behaviour.

### 3.2. Daily symptoms

Mean daily intrusions were significantly higher during pre-i-TFT (*B* = 0.89, *p* < .001) and i-TFT (*B* = 1.40, *p* < .001) than post-i-TFT (Figure 3; Supplementary Table S2). Post-i-TFT intrusions showed a significant negative slope over days (*B* = −0.06, *p* = .048), whereas slopes did not differ significantly between periods (all *p* > .05). Mean avoidance symptoms did not differ across periods (all *p* > .05). Negative mood and cognition were lower pre-i-TFT than both i-TFT (*B* *=* −0.61, *p* = .037) and post-i-TFT (*B* *=* −0.47, *p* = .020), indicating elevated negative mood and cognition symptoms during and after treatment. Arousal was lower in the pre-i-TFT period than post-i-TFT (*B* = −0.40, *p* = .048), reflecting increased arousal following treatment. Slopes for avoidance, negative mood and cognition, and arousal were not significant within and between periods (all *p* > .05).

**Figure 3.**
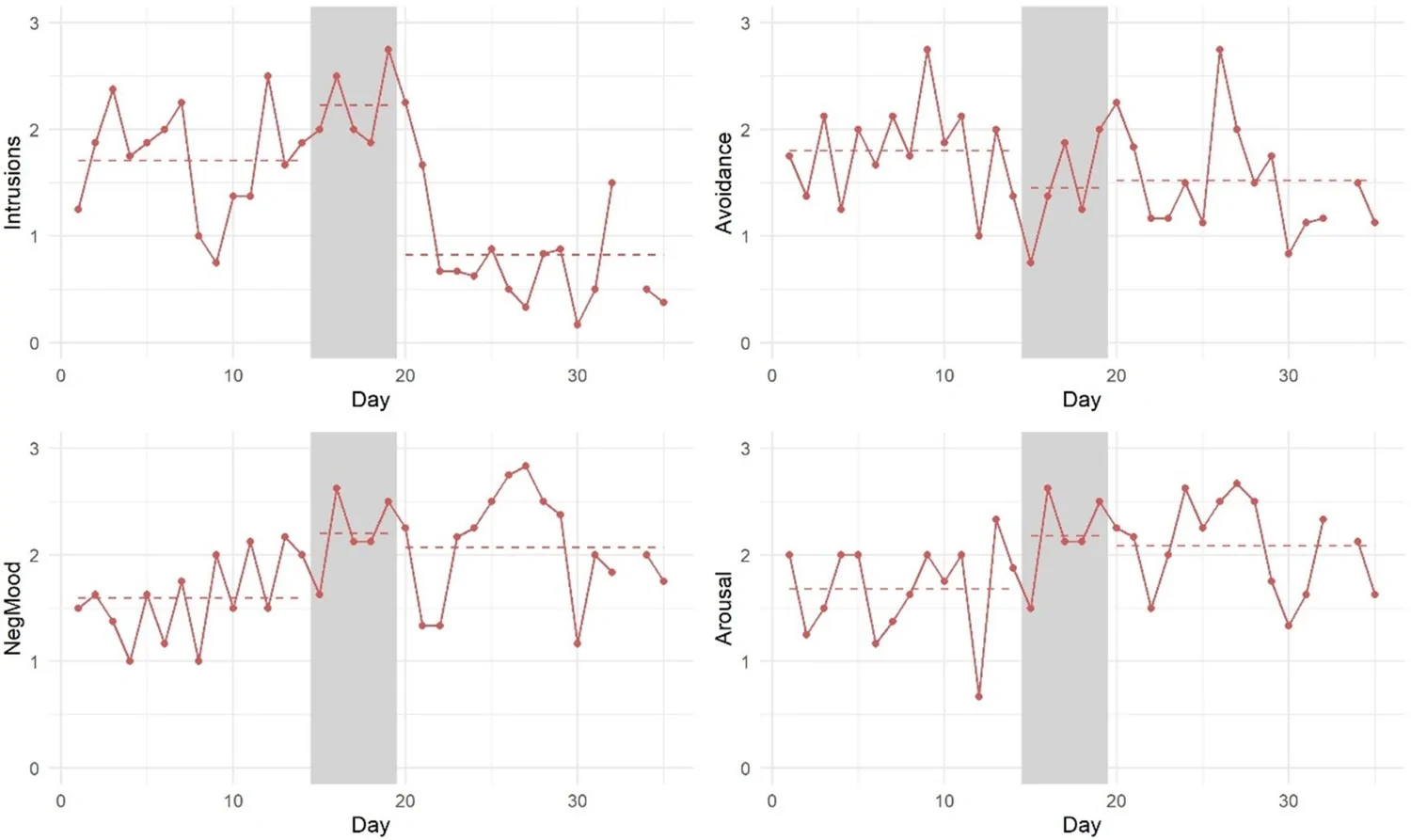
Time series of daily and period means of adolescent-reported PTSD symptoms. Note: Grey boxes mark the i-TFT period. Dashed horizontal lines show the mean score per period.

### 3.3. Daily maternal behaviours

Mean levels of adolescent- and mother-reported unnecessary helping remained stable across periods, with nonsignificant slopes within and between periods for both reporters (all *p* > .05; Figure 4). Mean adolescent-reported excessive monitoring did not differ between periods, but showed a significant negative slope post-i-TFT (*B* = −0.15, *p* = .038), which differed from the pre-i-TFT slope (*B* = −0.29, *p* = .012). Mother-reported excessive monitoring decreased slightly from pre-i-TFT to i-TFT, then increased at follow-up, with significant differences between post-i-TFT and both pre-i-TFT (*B* = 1.60, *p* = .001) and i-TFT (*B* = 2.02, *p* = .003), although slopes were nonsignificant. For cautious warnings, adolescent ratings increased significantly during pre-i-TFT (*B* = 0.19, *p* = .012), while mean mother ratings increased significantly from pre-i-TFT to post-i-TFT (*B* = −0.88, *p* = .024) and from i-TFT to post-i-TFT (*B* = −1.22, *p* = .026). All other effects were nonsignificant.

**Figure 4.**
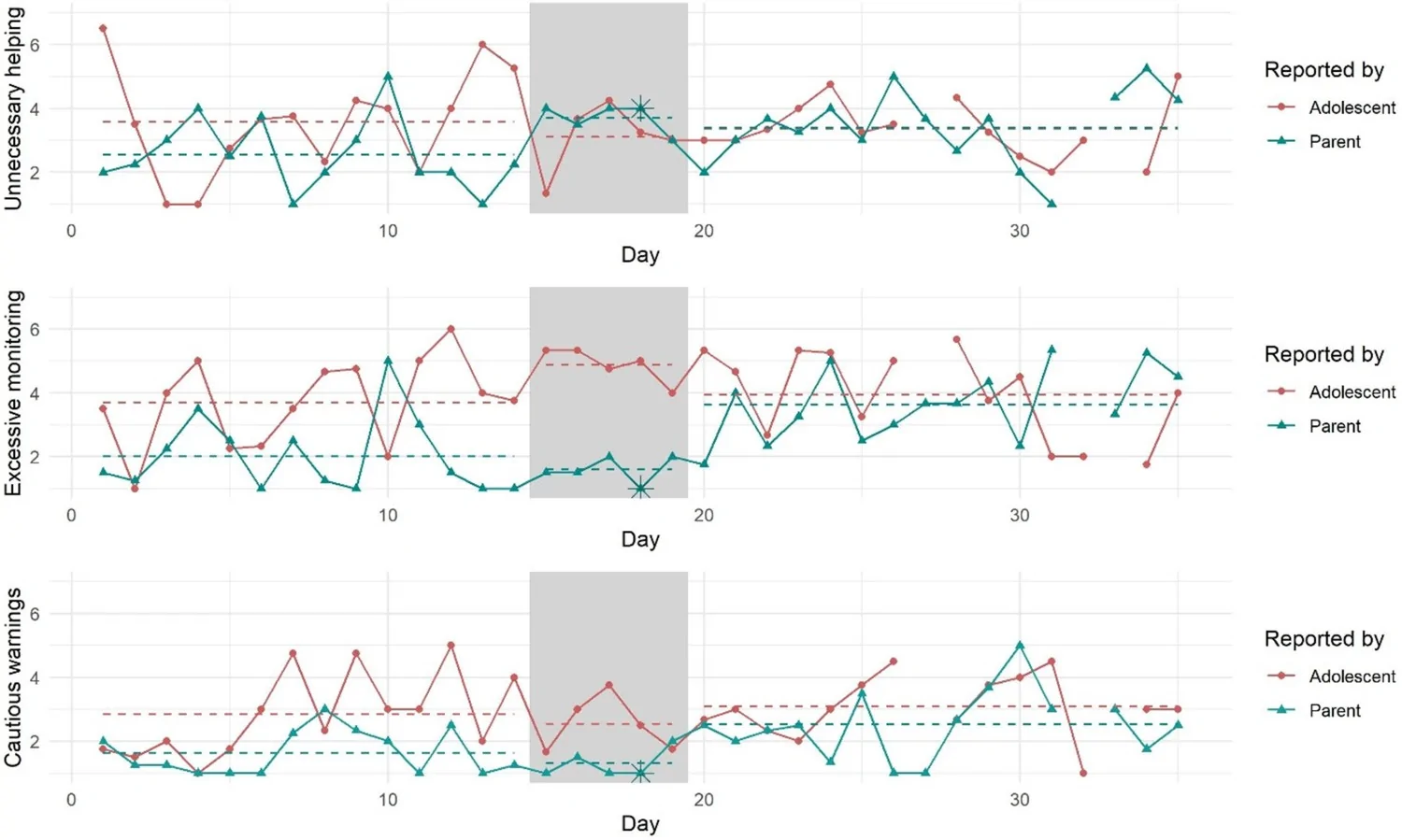
Time series of daily and period averages of maternal behaviours reported by the adolescent and the parent. Note: Grey boxes indicate the i-TFT period. Dashed horizontal lines represent the mean score for each period. The blue asterisks on day 18 is explained in Box 2.

Box 2. In the heat of the moment

During the break of day 4 of the i-TFT, the therapist returned to the break room without the adolescent. The mother showed signs of increased tension, including facial flushing, raised voice, and standing up. The therapist explained that the adolescent wanted time alone and had informed her mother via a text message. Despite this, the mother repeatedly asked to see her daughter and requested the therapist to take her to her. A few minutes later, the mother returned visibly upset; the adolescent had preferred to remain alone.

During the subsequent interview, the mother recalled*: ‘My panic … I immediately started running towards [daughter]’*. She reflected that the following day, the parent trainer introduced a pause-and-orient method to support adolescents, which she realised she could apply herself to remain calmer in stressful situations.

On day 4 of the i-TFT (asterisk in Figure 4), the mother reported relatively low levels of excessive monitoring, despite observed behaviour suggesting greater involvement (Box 2).

### 3.4. Different perspectives on parenting behaviours

Agreement between adolescent- and mother-reported perceptions on the three items related to parental overprotection and anxious rearing was minimal (unnecessary helping: *ρ* = 0.08, *p* = .475; excessive monitoring: *ρ* = −0.07, *p* = .547; cautious warning: *ρ* = −0.01, *p* = .921). During baseline, the adolescent reported higher levels than the mother on all three items. Over time, discrepancies between mother and daughter decreased for unnecessary helping and excessive monitoring, indicating some convergence, whereas cautious warnings diverged during i-TFT before converging again at follow-up.

Correlations between the difference scores and daily PTSD symptoms revealed significant negative associations. Specifically, higher mother-reported excessive monitoring relative to the adolescent was associated with fewer intrusions (*ρ* = −0.33, *p* = .002) and higher mother-reported cautious warnings were associated with fewer negative mood and cognition symptoms (*ρ* = −0.28, *p* = .011).

### 3.5. Momentary associations between parenting and adolescents’ PTSD symptoms

Autocorrelations indicated that intrusions (*ρ* = .31*, p* = .001) and negative mood and cognition (*ρ* = .28, *p* = .002) were significantly correlated with their own scores at the subsequent prompt. Among parenting behaviours, only mother-reported excessive monitoring showed significant short-term stability (*ρ* = 0.35, *p* = .004).

Contemporaneous correlations indicated that higher mother-reported excessive monitoring was associated with lower intrusions (*ρ* = −0.33, *p* = .001), whereas higher adolescent-reported cautious warnings were associated with greater negative mood and cognitions (*ρ* = 0.27*, p* = .007).

Cross-lagged correlations indicated that higher mother- and adolescent-reported cautious warnings at time *t* were associated with lower adolescent intrusions at time *t* *+* *1* (*ρ* = −0.23, *p* = .018; *ρ* = −0.24, *p* = .027, respectively). Additionally, higher adolescent-reported avoidance at time *t* was associated with greater adolescent-reported excessive monitoring at *t* *+* *1* (*ρ* = 0.22, *p* = .043). In contrast, higher intrusions at time *t* were associated with lower mother-reported excessive monitoring (*ρ* = −0.29, *p* = .030). No other significant cross-lagged associations were observed. Full results are presented in Supplementary Table S3.

### 3.6. Reflections on the parent training and EMA

During the interview, the mother reported that the parent training and EMA had helped her to reflect more on her own tension, fears, and the intentions behind her actions. She reported difficulty distinguishing between her behaviours towards her daughter that were genuinely helpful and those that were controlling, noticing that some actions were driven more by her own insecurities or anxieties than by her daughter’s needs (Box 3).

Box 3. Kind or controlling

The mother described: ‘*I bring her everything, so to speak, and I keep track of time … sometimes I feel like I am actually doing things that she could do herself.’* She added: *‘On Monday, it was tidying up, or helping her remember things … about school, for example. Sometimes it’s helping kindly, and sometimes it’s helping in a controlling way.’* She reflected: *‘Or sometimes it’s really too much because it comes from my own issues and doesn’t do anything good for [daughter] … I really do it clumsily, based on my own needs rather than focusing on her.’*

The mother also reflected on exercises from the parent training, which helped her become more aware of her own tension and its impact of her actions toward her daughter (Box 4). She reported that these experiences increased her awareness and confidence in supporting her daughter while managing her own anxieties.

Box 4. Reflections on the parent training

The mother reflected: *‘I thought the balloon exercise was very powerful.’* In this exercise, one person holds a balloon with their eyes closed while a co-regulator (another parent) provides verbal guidance to prevent startle responses when a pin touches the balloon. She continued: *‘You also notice that with a co-regulator, the subtitling exercise, the fact that it made such a difference to my physical reaction, was a special experience for me’*.

## 4. Discussion

This single-dyad study illustrates how multiple assessment methods can complement one another in capturing daily parent-child interactions during trauma-focused therapy. Using parental overprotection and anxious rearing as illustrative behaviours, we highlight the added value of combining surveys, EMA, observations, interviews, and multi-informant perspectives to gain a more comprehensive understanding of parent–child dynamics in adolescent PTSD treatment.

### 4.1. The benefit of daily parent and adolescent assessments

Intensive repeated measures such as EMA offer distinct advantages over retrospective surveys in the context of trauma-focused therapy. While surveys suggested a general decline in adolescent PTSD symptoms, particularly avoidance, leading to the conclusion that the adolescent no longer met criteria for PTSD after the i-TFT, EMA revealed a more nuanced pattern: intrusions increased during therapy before decreasing, avoidance remained stable, and negative mood, cognitions, and arousal, increased over time. This increase may reflect heightened emotional activity following trauma-focused treatment. According to the emotional processing theory, engaging with trauma-related memories during treatment, for example through exposure therapy, activates trauma-related fear structures while also reducing avoidant behaviour, which can lead to temporary increases in distress and arousal during and immediately following treatment (Foa & Kozak, 1986). This observation is also consistent with recent single-case findings showing temporary increases in daily PTSD symptoms during and after exposure therapy for some clients (Kooistra et al., 2026). Taken together, this finding indicates that conclusions drawn from retrospective surveys may differ from those based on EMA, which captures moment-to-moment fluctuations and transient distress that are often invisible in hindsight. This observation aligns with prior research showing that retrospective reports frequently reflect how symptoms are remembered rather than how they are experienced (Russell & Gajos, 2020; Schuler et al., 2021).

Beyond symptom monitoring, daily assessments can illuminate bidirectional parent-child influences (Keijsers et al., 2022). In this dyad, adolescent avoidance symptoms were associated with higher levels of adolescent-reported excessive maternal monitoring at the subsequent assessment, suggesting that specific PTSD symptoms may elicit specific parental behaviours. Conversely, maternal cautious warnings were linked to fewer intrusions at later assessments, indicating that some parental behaviours may exert short-term protective effects. Although these findings should not be interpreted as evidence for general relationships between parenting behaviours and PTSD symptoms, they illustrate how intensive repeated measures within trauma-focused treatment can reveal reciprocal interactions that fuel or dampen children’s symptoms within an individual family. These dynamics, that are often missed by traditional methods, may foster a more systemic understanding of adolescent psychopathology and provide valuable input for innovative, personalised clinical interventions.

### 4.2. Understanding parenting through diverse viewpoints

Integrating both parent and adolescent perspectives provided additional insights into daily parenting and its interaction with adolescent PTSD. Consistent with prior research, agreement between parent- and adolescent-reported parenting behaviours was minimal (e.g. De Los Reyes et al., 2019). The mother reported lower levels of unnecessary helping, excessive monitoring, and cautious warnings than her daughter, reflecting a more positive self-perception (Bögels & van Melick, 2004). These discrepancies highlight the importance of including reports from both informants, as associations with symptoms may vary depending on the reporter. In line with previous findings (e.g. Janssen, Verkuil, et al., 2021), the discrepancies themselves were also linked to symptoms: greater overprotection reported by the mother relative to the daughter was associated with fewer intrusions and less negative mood and cognition, suggesting that behaviours not perceived by the adolescent as overly controlling may have protective effects.

At follow-up, discrepancies between mother- and daughter-reported parenting had decreased, a change further illuminated by the interview. The mother described increased awareness of her own tension and parenting behaviours, likely fostered by the parent training, which included psychoeducation and reflective exercises. This kind of self-reflection may have helped her evaluate her behaviour in ways more consistent with her daughter’s experiences.

Observations further contextualised the survey and EMA data by providing real-life examples of behaviours reported in the survey, such as the mother making decisions for her daughter. They also captured overprotective behaviours that EMA did not detect – for instance, the mother insisting on accompanying her daughter despite her wish to be alone. Taken together, the use of multiple informants, observations, and interviews offers a more comprehensive understanding of parenting, revealing intentions, context, and interpretations that would be missed by EMA or questionnaires alone.

### 4.3. Challenges, recommendations and future directions

Multi-method, multi-informant longitudinal studies are highly valuable, as they can uncover family dynamics that may remain hidden with traditional measures. Nevertheless, it also presents several practical challenges. First, participation demands considerable time and effort from families (Russell & Gajos, 2020). Families undergoing trauma therapy may already feel overwhelmed, which can reduce questionnaire completion and overall compliance. Low EMA compliance among other participating dyads in this study appeared to be related to a lack of motivation stemming from insufficient direct feedback, missed EMA prompts due to school activities or increased sleep, or an increase in symptoms. These challenges highlight the importance of designing EMA protocols that are both feasible and engaging for clinical populations. Providing feedback or brief personalised updates throughout the assessment period may help maintain motivation (Bülow et al., 2025). However, such approaches may also influence participants’ responses and should therefore be carefully considered. Second, implementing and conducting EMA in clinical practice is time-intensive for clinicians, potentially limiting its feasibility in routine settings. Third, single-dyad (*N* = 2) study designs present unique ethical dilemmas. In *N* = 1 research, it is common practice to provide participants with the manuscript before publication allowing them to view and approve the manuscript. However, this process is more complex in *N* = 2 research, as multiple participants may have differing perspectives on shared experiences (De Los Reyes & Kazdin, 2005), particularly when these experiences involve sensitive information related to parenting and trauma-related symptoms. Asking approval beforehand from both participants to access each other’s responses may introduce response and selection biases, as participants may alter their responses or withdraw consent when confronted with sensitive information about themselves or their relationship. Conversely, not explicitly informing participants that the study focuses on their dyad also raises ethical concerns, as they may identify themselves and the information provided by the other person in a published paper. Researchers should therefore either obtain explicit consent for data sharing while carefully considering potential biases or sufficiently anonymize the data to prevent identification.

Future research should aim to develop methods that provide meaningful, manageable data while minimising the burden for clinicians and patients. This may be achieved by optimising assessment protocols, for example by reducing the number of prompts, using shorter EMA protocols, and identifying the most informative assessment moments and measures to balance between feasibility and data richness. Integrating EMA into routine clinical care may also facilitate its implementation and use in clinical practice. Moreover, incorporating qualitative open-ended questions within EMA may provide sufficient contextual information to reduce the need for additional interviews. Such optimised designs may enable replication in larger studies, allowing researchers to evaluate the generalizability of associations between parenting and adolescent PTSD. Beyond its use as an assessment method, future studies should also explore the usefulness of EMA as an intervention tool, for example by enhancing patients’ awareness of triggers, symptoms, and coping strategies.

### 4.4. Strengths and limitations

This study has notable strengths. These include the use of a multi-method, multi-informant approach within a clinical setting and the high compliance of both mother and daughter. This combination enabled capturing within-person fluctuations, contextual details, and participants’ interpretations, providing a rich and nuanced understanding of parent-child interactions.

Several limitations should also be acknowledged. First, not all EMA items were validated for frequent daily assessments, and some, such as avoidance, are inherently difficult to recognise in real time, as they involve actions not taken rather than directly observable behaviours. Second, since the father and sibling did not participate, this limits insights into the full family dynamics and their perspectives, and how these link with adolescent symptoms. Third, the naturalistic observations were exploratory, and as such they were not recorded and not independently double-coded using a standardised coding scheme. Therefore, observer bias cannot be ruled out and the reliability and reproducibility of these observations are limited. Finally, the study’s findings are limited to a single dyad. The observed patterns are specific to this mother–daughter dyad and their relationship dynamics and trauma history. Therefore, the findings should be interpreted within the context of this specific family and not be generalised to other families.

### 4.5. Clinical implication and conclusion

This single-case study on the role of maternal overprotection demonstrates that combining surveys, EMA, observations, interviews, and both parent and adolescent perspectives provides a detailed view of daily maternal behaviour in relation to a child’s PTSD during trauma-focused therapy. Real-time symptom tracking allows therapists and patients to identify periods of heightened or reduced distress and explore potential protective factors or triggers, enabling targeted interventions that may enhance treatment outcomes. Although the specific findings from this study are based on one mother–daughter dyad and should be interpreted within this specific family context, the multi-method, multi-informant approach may provide a valuable framework for examining daily dyadic processes also in other contexts, such as sibling relationships or coparenting during divorce. Clinicians and researchers should be cautious about relying on a single informant or assessment method and instead incorporate multiple perspectives to obtain accurate insights into dyadic interactions. At the same time, these approaches are demanding, highlighting the need for future work to identify clinically relevant and efficient measures, minimise participant burden, and implement strategies to enhance participant engagement.

## Supplementary Material

EJPT_Supplementary Materials.docx

## Data Availability

The data that support the findings of this study are not publicly available due to the sensitive nature of the data and the small number of participants. De-identified data may be obtained from the first author upon reasonable request.
