# Supplementary material for: Helping or controlling? A multi-method case study of a mother-daughter dyad and their interactions during trauma-focused therapy
Source: Eur J Psychotraumatol. 2026 Sep 28;17(1):2731853. doi: 10.1080/20008066.2026.2731853 (PMC13629806; doi:10.1080/20008066.2026.2731853)
Supplement: Supplementary file 1 [file ZEPT_A_2731853_SM1269.docx]

Supplementary Materials

**S1. Intensive trauma-focused treatment**

***Program***

|  | **Adolescent** | **Parent** |
| --- | --- | --- |
| 9:45 am – 10:00 am | Start of the day: activity | |
| 10:00 am – 11:00 am | Exposure therapy | Psychoeducation |
| 11:00 am – 11:15 am | Break in common room | |
| 11:15 am – 12:15 am | EMDR therapy | Psychoeducation |
| 12:15 am – 12:30 am | Joint session of parent-child to share experiences | |
| 12:30 am – 1:00 pm | Physical activity | |

***Parent training***

| **Day** | **Modules** |
| --- | --- |
| Monday | 1. Introduction 2. Different types of trauma |
| Tuesday | 3. Understanding the impact of trauma 4. Creating a safe environment place for children |
| Thursday | 5. Dealing with feelings and behaviors 6. Connection and recovery |
| Friday | 7. Becoming an advocate for your child 8. Taking care of yourself |

Each day began with a brief activity to facilitate social interaction among families. Between therapy sessions, a 15-minute break was held in a shared common room for both adolescents and parents. At the end of the therapy day, parents and adolescents reunited in the therapy room, offering an opportunity for the adolescent to share their experiences of that day. Each day ended with 30 minutes physical activity involving all families, during which families chose their preferred form of exercise (e.g., playing soccer, tossing a ball, walking).

**Table S1**

*CATS-2 scores*

| DSM-5 Criterion | Total score | | Number of symptoms | | Required symptoms |
| --- | --- | --- | --- | --- | --- |
|  | Baseline | Follow-up | Baseline | Follow-up |  |
| Intrusions  Items 1-5 | 9 | 6 | 3 | 2 | 1+ |
| Avoidance  Items 6-7 | 6 | 2 | 2 | 0 | 1+ |
| Negative mood & cognition  Items 8-14 | 10 | 10 | 4 | 3 | 2+ |
| Arousal  Items 15-20 | 14 | 9 | 5 | 2 | 2+ |

| **Table S2**  *Mean (SD) symptom levels per period* | | | | |
| --- | --- | --- | --- | --- |
|  |  | Pre i-TFT | i-TFT | Post i-TFT |
| Symptoms | |  |  |  |
|  | Intrusions | 1.71 (0.51) | 2.23 (0.38) | 0.82 (0.57) |
|  | Avoidance | 1.80 (0.45) | 1.45 (0.50) | 1.52 (0.52) |
|  | Negative mood & cognitions | 1.60 (0.39) | 2.20 (0.39) | 2.07 (0.51) |
|  | Arousal | 1.68 (0.45) | 2.17 (0.44) | 2.08 (0.43) |
| Adolescent-reported behavior | |  |  |  |
|  | Unnecessary helping | 3.57 (1.67) | 3.10 (1.10) | 3.35 (0.91) |
|  | Excessive monitoring | 3.70 (1.39) | 4.88 (0.55) | 3.94 (1.39) |
|  | Cautious warnings | 2.85 (1.32) | 2.53 (0.88) | 3.08 (0.97) |
| Mother-reported behavior | |  |  |  |
|  | Unnecessary helping | 2.55 (1.11) | 3.70 (0.45) | 3.38 (1.16) |
|  | Excessive monitoring | 2.02 (1.19) | 1.60 (0.42) | 3.62 (1.12) |
|  | Cautious warnings | 1.63 (0.69) | 1.30 (0.45) | 2.52 (1.06) |

| **Table S3**  *Autocorrelations, contemporaneous and cross-lagged correlations of all main variables* | | | | | | | | | | | | | | | |
| --- | --- | --- | --- | --- | --- | --- | --- | --- | --- | --- | --- | --- | --- | --- | --- |
|  |  | |  | Intrusions | | | Avoidance | | | Negative mood and cognition | | | Arousal | | |
|  | | AC | | CC | TC_S 🡪 B_ | TC_B 🡪 S_ | CC | TC_S 🡪 B_ | TC_B 🡪 S_ | CC | TC_S 🡪 B_ | TC_B 🡪 S_ | CC | TC_S 🡪 B_ | TC_B 🡪 S_ |
| AC | |  | | **0.31**** | | | 0.16^†^ | | | **0.28**** | | | 0.14 | | |
| Adolescent-report | |  | |  |  |  |  |  |  |  |  |  |  |  |  |
|  | Unnecessary helping | 0.11 | | -0.07 | 0.04 | -0.01 | 0.06 | 0.01 | -0.12 | 0.16 | -0.03 | 0.19^†^ | 0.16 | 0.10 | -0.06 |
|  | Excessive monitoring | 0.04 | | 0.18^†^ | 0.14 | 0.00 | -0.05 | **0.22*** | -0.12 | 0.15 | 0.20^†^ | -0.03 | 0.11 | 0.10 | 0.14 |
|  | Cautious warning | 0.09 | | -0.07 | -0.02 | **-0.24*** | 0.09 | 0.08 | 0.20^†^ | **0.27**** | 0.04 | 0.09 | -0.09 | -0.17 | 0.01 |
| Mother-report | |  | |  |  |  |  |  |  |  |  |  |  |  |  |
|  | Unnecessary helping | 0.03 | | -0.16 | -0.04 | -0.03 | -0.09 | -0.04 | -0.03 | -0.10 | 0.10 | 0.13 | 0.04 | 0.04 | 0.14 |
|  | Excessive monitoring | **0.35**** | | **-0.33**** | **-0.29**** | -0.19^†^ | -0.03 | -0.04 | -0.04 | 0.12 | 0.06 | 0.15 | 0.16 | 0.05 | 0.10 |
|  | Cautious warning | -0.11 | | -0.12 | -0.14 | **-0.23*** | -0.05 | -0.04 | -0.04 | -0.11 | 0.14 | 0.18^†^ | -0.19^†^ | 0.00 | 0.01 |

*Note.* AC = autocorrelations. CC = contemporaneous correlations. TC = temporal correlations. S 🡪 B = Symptoms at time *t* and behavior at time *t+1*. B🡪S = behaviors at time *t* and symptoms at time *t +1*. ** p <0.01, *p <0.05, ^†^p<0.10
